# Draft Genome Sequence of Aryl Hydrocarbon Receptor Activator Strains Lactobacillus reuteri R2lc and 2010

**DOI:** 10.1128/MRA.00067-19

**Published:** 2019-04-04

**Authors:** Mustafa Özçam, Jan-Peter van Pijkeren

**Affiliations:** a Department of Food Science, University of Wisconsin—Madison, Madison, Wisconsin, USA; Queens College

## Abstract

Lactobacillus reuteri R2lc and 2010 are pigmented rat intestinal isolates. L. reuteri R2lc has been studied in different animal disease models, including colitis and acute liver injury. We sequenced both isolates and identified novel secondary metabolite gene clusters, which may be linked to the probiotic potential of these strains.

## Abstract (precis)

Lactobacillus reuteri R2lc and 2010 are pigmented rat intestinal isolates. L. reuteri R2lc has been studied in different animal disease models, including colitis and acute liver injury.

## ANNOUNCEMENT

Probiotics are defined as live microorganisms which, when administered in adequate amounts, confer a health benefit on the host (1). Select Lactobacillus reuteri strains activate the aryl hydrocarbon receptor (AhR)—a ligand-activated transcription factor that modulates the immune system (2)—through tryptophan metabolism, which protects against *Candida* infection (3). We recently showed that two pigmented L. reuteri strains, R2lc and 2010, activate AhR (4). L. reuteri R2lc is a probiotic candidate with anti-inflammatory properties and has been studied in different disease models (5–11). Here, we report the draft genome sequences of L. reuteri strains R2lc and 2010.

L. reuteri R2lc and 2010 were isolated from rat gastrointestinal tracts in Lund, Sweden (∼1990), and Raleigh, NC (∼1988), respectively. The glycerol stocks of L. reuteri strains were inoculated in De Man-Rogosa-Sharpe (MRS) medium (Difco, BD BioSciences) and incubated at 37°C. The cultures were plated on MRS agar to isolate single colonies, which were inoculated in MRS broth followed by 16 h incubation. Genomic DNAs were isolated with the genomic DNA purification kit (Wizard, Promega). DNA concentrations were measured with the Qubit double-stranded DNA (dsDNA) high-sensitivity assay kit (Life Technologies). Genome sequencing was performed at the Biotechnology Center of the University of Wisconsin—Madison. Samples were prepared using a TruSeq Nano DNA low-throughput (LT) library prep kit (Illumina, Inc.) according to the manufacturer’s instructions with small modifications. The quality and quantity of the finished libraries were analyzed using an Agilent DNA 1000 chip and Qubit dsDNA high-sensitivity (HS) assay kit. DNA libraries were standardized to 2 nM. Paired-end (250-bp) sequencing was performed using the Illumina MiSeq sequencer and a MiSeq 500-bp version 2 sequencing cartridge. Images were analyzed using the standard Illumina Pipeline version 1.8.2. The final output yielded 1,005,752 and 1,010,053 raw reads with 504,887,504 and 507,046,606 total bases for R2lc and 2010, respectively. Quality control and *de novo* assemblies of sequence reads were performed with the SeqMan NGen software package version 12.3.1.4 (DNAStar) using standard settings. The plasmid genomes were closed by primer walking. We annotated the genomes with the NCBI Prokaryotic Genome Annotation Pipeline (12).

The draft genomes of R2lc and 2010 have a predicted size of 2,084,790 (*N*_50_ value of 132,336 bp with 58 contigs) and 2,214,494 (*N*_50_ value of 193,364 bp with 38 contigs) base pairs, GC contents of 38.45% and 38.52%, and 2,128 and 2,158 open reading frames, respectively. Genes unique to R2lc and 2010 were identified with the phylogenetic gene profiler that is embedded in the Integrated Microbial Genomes and Microbes (IMG/M) data set of the Joint Genome Institute (JGI) (13). The presence of secondary metabolite gene clusters was predicted with antiSMASH (14). These analyses revealed that R2lc putatively encodes two distinct polyketide synthase (PKS) gene clusters (*pksR2lc01* and *pksR2lc02*), each encoded from a plasmid. Strain 2010 encodes a single PKS cluster (*pks2010*). With BLASTP analyses, we determined that the *pks2010* cluster is homologous to *pksR2lc02*, with amino acid identities ranging from 53 to 87% (4).

### Data availability.

The genome sequences of L. reuteri R2lc and 2010 have been deposited in NCBI GenBank under the accession numbers PTLS00000000 and PUXG00000000, respectively. The original raw sequence data of L. reuteri R2lc and 2010 can be found at the NCBI Sequence Read Archive under the accession numbers SRR8165042 and SRR8165043, respectively.
